# Perioperative considerations for peripheral nerve stimulation devices: A practical guide^☆^

**DOI:** 10.1016/j.inpm.2025.100628

**Published:** 2025-08-11

**Authors:** Stefani M. Schwartz, David Hao

**Affiliations:** Department of Anesthesia, Critical Care and Pain Medicine, Massachusetts General Hospital, Harvard Medical School, Boston, MA, USA

**Keywords:** Neuromodulation, Implanted devices, Peripheral nerve

## 1 Introduction

Peripheral nerve stimulators (PNS) are increasingly encountered in the perioperative setting as clinical indications continue to expand [[1], [2], [3], [4]]. Compared to spinal cord stimulators, where perioperative management is supported by more established guidelines, PNS guidance remains limited and is often reliant on device-specific manuals [5,6]. This editorial provides a concise, stepwise guide to assist in the safe and effective management of patients with PNS in the perioperative setting. Device manufacturers may revise recommendations over time and new technologies continue to emerge; therefore, we recommend that clinicians remain aware of evolving guidelines.

**Step 1: Identify the Device Early**

**Fig. 1:**
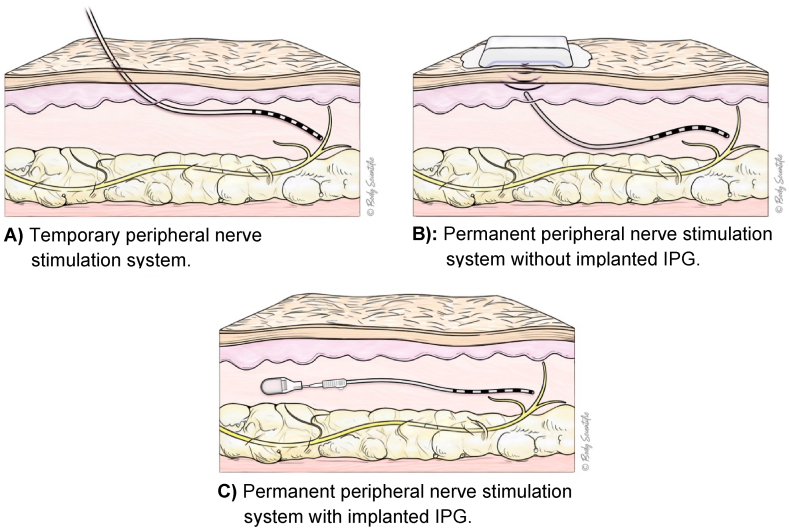
PNS system variants: Lead and generator configurations.

Early identification of a PNS is essential and should occur prior to the day of surgery, ideally at the time of the preoperative evaluation (see Table 1, Fig. 1). Given the wide range of systems [7] and the variation in design and location, it is especially important to document specific details, including [5]:

• Type of PNS device and indication
• Location of electrodes and, if applicable, the implanted pulse generator (IPG)
• External control method (e.g., personal phone or manufacturer-supplied remote)
• Current status (on or off)
• Manufacturer and local representative contact information

**Table 1:** Peripheral nerve stimulation systems.

Device Name (Manufacturer)	Temporary or Permanent Electrode	Pulse Generator	Patient Controller
Sprint PNS System (SPR)	Temporary (up to 60 days)	External (adhesive mounting pad)	Hand-held remote
Freedom Peripheral Nerve Stimulator System (Curonix)	Permanent	External (wearable transmitter assembly)	Transmitter remote
StimRouter Neuromodulation System (Bioventus)	Permanent	External (adhesive mounting pad)	Patient programmer remote
Nalu Neurostimulation System (Nalu)	Permanent	Internal (implantable pulse generator)	Therapy disc or smartphone-based app

Early coordination with the implanting team, pain management specialist, and/or device representative is optimal to address any device-specific considerations as delayed recognition may lead to increased perioperative risk. Patients should also be counseled that, even when precautions are followed, there remains a risk of device failure, or need for device revision or explanation.

**Step 2: Turn the Device Off**

It is generally considered standard practice to turn off neuromodulation devices before surgery or procedures to reduce the risk of thermal injury and avoid signal interference [5]. While not all PNS manufacturers explicitly state this, the rationale is consistent across platforms. For example, SPRINT (SPR) warns that the use of high-frequency surgical equipment may cause burns at the electrode tip or mounting pad, but does not clearly instruct the device be turned off [8]. StimRouter (Bioventus) advises against using electrosurgical tools near the lead, citing risk of unintended stimulation and severe injury [9]. Freedom PNS (Curonix) offers electrocautery precautions without direct instructions to deactivate the device [10]. Only Nalu explicitly recommends turning off stimulation prior to procedures [11].

Because SPRINT (SPR) is intended for removal at 60 days, it is reasonable to discuss whether early removal is appropriate before surgery. For other systems lacking specific guidance, turning off the device remains a prudent step based on consensus recommendations across neurostimulation technologies.

**Step 3: Position Carefully**

Patient positioning should be discussed preemptively with the surgical team. Understanding the location of the lead and, if applicable, the IPG is essential to avoid pressure-related or mechanical injury to the device. For new implants placed within the past 6 weeks, extra care should be taken to position limbs neutrally to prevent lead migration.

While there are no formal manufacturer guidelines regarding heat exposure, it may be reasonable to avoid prolonged direct contact with heating devices such as Bair Hugger systems and underbody water blankets.

**Step 4: Electrocautery Precautions**

Electrocautery poses a risk of thermal injury at the lead or IPG site. When feasible, bipolar electrocautery is preferred across all devices and is considered the safest option. If monopolar cautery is necessary, precautions should include using the lowest effective current delivered in short bursts (“low voltage, short bursts”). The return pad should be placed as far away from the IPG as possible, ideally on the contralateral side of the lead and/or IPG. Current paths across the implantable device should be avoided [5].

Manufacturer-specific guidance supports these general principles. Nalu explicitly recommends against the use of monopolar cautery [11]. SPRINT (SPR) labeling warns of burn risk at the lead or mounting pad during concurrent electrocautery use [8]. Freedom PNS (Curonix) permits monopolar cautery with appropriate precautions, including use of low-voltage modes and distant placement of the return pad [10]. Across platforms, the overarching recommendation is to minimize the risk of unintended current transfer through the implanted system.

**Step 5: Regional Anesthesia, Caution, Not Contraindication**

Regional anesthesia may be used as primary anesthesia or as an adjunct for analgesia. However, careful planning is essential to prevent lead displacement or shearing. Particular attention should be paid to the location of the electrode and all associated components to ensure they are not disrupted during regional techniques. Patients who receive regional anesthesia may experience a temporarily altered response to stimulation or encounter high impedance warnings on their devices. We recommend keeping the device turned off for the expected duration of the block.

**Step 6: Postoperative Device Reactivation**

Postoperatively, patients and caretakers should be aware that delivering stimulation near a surgical incision may interfere with healing. The SPRINT (SPR) labeling specifically advises against use of stimulation over or near surgical incisions [8].

If the PNS is located sufficiently far from the surgical site, the device may be turned back on in the immediate postoperative period once the patient has recovered from anesthesia and it is deemed clinically appropriate. Patients may self-direct this process, provided it is determined that resuming therapy is safe. The pain medicine service or device representative may be contacted to assist with device interrogation and restoration of function, if needed. If the patient reports changes in stimulation or if interrogation reveals abnormal function, the device should be evaluated promptly by the implanting team or manufacturer representative.

## 2 Other procedural/surgical contraindications

Most manufacturers recommend against the use of diathermy, external defibrillation, laser therapy, radiation therapy, and dental drills in patients with a PNS, as these interventions may cause serious bodily harm or damage to the implantable device [5,[8], [9], [10], [11]]. If any of these interventions are medically necessary, it is advisable to consult a pain medicine specialist.

## Declaration of competing interest

The authors declare that they have no known competing financial interests or personal relationships that could have appeared to influence the work reported in this paper.
